# Alcohol-attributable burden of cancer in Argentina

**DOI:** 10.1186/s12889-022-12549-7

**Published:** 2022-01-18

**Authors:** I A T van de Luitgaarden, A E Bardach, N Espinola, I C Schrieks, D E Grobbee, J W J Beulens

**Affiliations:** 1 University Medical Center Utrecht, Utrecht University, Julius Global Health, Julius Center for Health Sciences and Primary Care, Huispost Str. 6.131, 3508 GA Utrecht, the Netherlands; 2 Institute for Clinical Effectiveness and Health Policy, Buenos Aires, Argentina; 3 National Scientific and Technical Research Council - Argentina (CONICET), Buenos Aires, Argentina; 4 Julius Clinical, Zeist, the Netherlands; 5 Department of Epidemiology and Biostatistics, Amsterdam University Medical Center Location VUmc Amsterdam Cardiovascular Sciences Research Institute, Amsterdam, the Netherlands

## Abstract

**Introduction:**

Alcohol consumption is a risk factor for several types of cancer. Alcohol consumption levels in Argentina are among the highest in the world, and malignant neoplasms are the second cause of death in the country. Public health strategies aimed at reducing alcohol consumption could possibly lead to a decrease in cancer burden. Alcohol-attributable burden has been estimated before in neighboring countries Chile and Brazil. We now aimed to quantify the burden for Argentina.

**Methods:**

We obtained data on alcohol consumption levels from a national representative health survey and etiologic effect sizes for the association between alcohol and cancer from the most recent comprehensive meta-analysis. We estimated the number of alcohol-attributable cancer-related deaths and disability-adjusted life years (DALYs), stratified by consumption level (light (0.1–12.5 g/day), moderate (12.6–50 g/day), or heavy (> 50 g/day) drinking). We additionally explored which hypothetical scenario would achieve the highest reduction in alcohol-attributable cancer burden: 1) heavy drinkers shifting to moderate drinking or 2) moderate drinkers shifting to light drinking.

**Results:**

In 2018, 53% of the Argentinean population consumed alcohol. In men 3.7% of all cancer deaths and DALYs were attributable to alcohol consumption, in women this was 0.8% of all cancer deaths and DALYs. When moderate drinkers would shift to light drinking, 46% of alcohol-attributable cancer deaths and DALYs would be prevented, opposed to only 24% when heavy drinkers would shift to moderate drinking.

**Conclusion:**

Most cancer deaths and DALYs were attributable to moderate alcohol consumption (50%). This calls for implementation of population-wide strategies—instead of targeting heavy drinking only—to effectively reduce harmful use of alcohol and its impact on disease burden.

**Supplementary Information:**

The online version contains supplementary material available at 10.1186/s12889-022-12549-7.

## Introduction

Alcohol consumption belongs to the top ten risk factors for disease worldwide [1]. Alcohol consumption is associated in a dose-responsive manner with a higher risk for several diseases, such as several types of cancer and liver cirrhosis, as well as vehicle accidents and (un)intentional injury [2–5]. On the other hand, moderate alcohol consumption is thought to have potential benefits on ischemic heart disease and diabetes [6, 7]. Therefore, most international guidelines on alcohol consumption allow for a limited consumption of alcohol but emphasize that complete abstention from alcohol remains best [8–10]. The public opinion on alcohol consumption in many countries is that it is culturally accepted as social habit, which encourages its use. Argentina is part of the region of Southern Latin America. This region has the highest per capita alcohol consumption in Latin America and consumption levels are also higher than in most other world regions [2]. Furthermore, the wine and beer industries have grown in the last decade and are very prominent in the region, particularly in some Andean provinces. Drinking is ingrained in the country’s culture and has increased during the COVID-19 pandemic [11]. However, observational data on alcohol consumption, as well as estimations of impact of alcohol consumption on society, are relatively scarce in this part of the world [12].

The main cause of death in Argentina is cardiovascular disease (28%) followed by cancer (20%), together accounting for 48% of annual mortality [13]. Age-standardized incidence and mortality rates of cancer in Argentina are among the highest on the continent. Compared to most high-income countries, incidence rates of cancer are lower in Argentina, but mortality rates are comparable. Therefore, the burden of disease attributable to cancer in Argentina is substantial. Breast cancer has the highest incidence and mortality rates in the country, followed by prostate, colorectal, and lung cancer [14].

There is consistent evidence for a detrimental association between alcohol consumption and six types of cancer: breast, esophageal, liver, oral cavity and pharynx, larynx, and colorectal cancer [15–17]. Several mechanisms are thought to play a role in the pathophysiology, including amongst others the direct toxic effect of acetaldehyde (the product of alcohol degradation), changes in hormone concentrations, and production of harmful reactive-oxygen species [18, 19]. Some mechanisms require substantial amounts of alcohol to be consumed, whilst others already occur with light-to-moderate consumption [17].

Worldwide, large differences in alcohol-attributable cancer burden exist between regions: most alcohol-attributable cancer deaths are found in the Western pacific region (7.8% of all cancer deaths), while in the Eastern Mediterranean region only 0.7% of all cancer deaths is attributable to alcohol. This percentage is 4% in the region of the Americas [20]. Alcohol-attributable burden of cancer has been recently described for Chile and Brazil [21, 22]: in both countries, alcohol consumption was estimated the third preventable cause of cancer incidence and mortality, accounting for 3.1% (in Chile) and 4.5% (in Brazil) of all cancer deaths. For Argentina specifically, alcohol-attributable burden of cancer has not yet been quantified.

The data from the previous studies in neighboring countries show the importance of regulating alcohol consumption and the need for public awareness on this topic. Moreover, it is of interest to know whether the prevention paradox [23] also applies to alcohol-attributable cancer burden, since this determines the type of public health strategy that would be most effective in reducing this burden. The aim of the present study was to estimate the impact of alcohol consumption on cancer burden in Argentina.

## Methods

All methods were carried out in accordance with relevant guidelines and regulations. We conducted a comparative risk assessment analysis, using as comparator a hypothetical scenario in which nobody would drink alcohol, to estimate the number of cancer deaths and DALYs that would be prevented.

### Calculation of population attributable fraction

We calculated the population attributable fraction (PAF) of alcohol using the formula below [24].

$$PAF=\frac{\sum_{i=1 }^{n}{P}_{i}\left({RR}_{i}-1\right)}{\sum_{i=1}^{n}{P}_{i}\left({RR}_{i}-1\right)+1}$$

where *n* is the number of exposure levels; in our case three (light, moderate, and heavy drinking), *P* is the proportion of the population at level i of exposure and *RR*_*i*_ is the relative risk of cancer at level i of exposure.

The PAF is the proportional reduction in disease or mortality that would occur if exposure to alcohol would be reduced to zero (i.e., no alcohol consumption), and thus is the proportion of disease/mortality that can be attributed to alcohol consumption. The main inputs to this formula are: 1) the etiological association (relative risk) between alcohol and site-specific cancer and 2) the current prevalence of alcohol consumption in Argentina.

### Risk estimates alcohol and site-specific cancer

We derived relative risks (RR) for different levels of alcohol consumption with site-specific cancer risk from the most comprehensive high-quality meta-analysis to date, conducted by Bagnardi et al. [17]. In this meta-analysis, sex-specific relative risks were reported for various alcohol consumption levels: non-drinkers, ≤ 12.5 g/day, 12.6—50 g/day, and > 50 g/day, in which the unit represents grams of pure alcohol consumed per day. Non-drinkers (including former drinkers) were regarded the reference category. Statistically significant associations with at least one level of alcohol consumption were reported for six types of cancer: breast, esophagus, liver, oral cavity and pharynx, larynx, and colorectal cancer. When associations were not statistically significant, we assumed the relative risk to be 1. All analyses were adjusted for age and relevant confounders specific for each cancer site (Supplementary Table 1).

### Prevalence of alcohol consumption

To estimate current prevalence of alcohol consumption levels we used data from the 4^th^ national risk factor survey, held in 2018 in Argentina. This is a nationwide representative survey of 29,224 individuals aged > 18 years from the general population [25], data are publicly available from: https://www.indec.gob.ar/indec/web/Institucional-Indec-BasesDeDatos-2. The survey contained a beverage-specific quantity-frequency questionnaire on alcohol consumption. Participants had to report their habitual weekly or, when drinking less than weekly, monthly consumption of units of beer, wine, and spirits. A standard unit in the Americas in general contains approximately 14 g of alcohol [9]. We calculated the total alcohol consumption per person in g per day. To be consistent with the consumption levels used in the meta-analysis, we categorized the participants based on their daily alcohol consumption into 1) non-drinkers, 2) light drinkers (0.1 – 12.5 g/day), 3) moderate drinkers (12.6 – 50 g/day), and 4) heavy drinkers (> 50 g/day). Similar categories were used for men and women. We accounted for complex survey design by using sampling weights. We presented the prevalence of the aforementioned consumption categories stratified by age (categorized into 18–29 y, 30–39, 40–49 y, 50–59 y, 60–69 y, and > 70 y) and sex.

### Calculation of deaths attributable to alcohol

We used vital registration data from the Directorate of Health Statistics and Information (DEIS) of the Argentine government to derive cause-specific mortality for the year 2018 [26], data are publicly available from https://www.argentina.gob.ar/salud/deis/datos. Data were coded according to the *International Classification of Diseases, Tenth Revision (ICD-10).* We selected the following codes: C50 (breast cancer), C18-20 (colorectal cancer), C15 (esophagus), C00-C14 (cancer of lip, oral, pharynx), C32 (larynx cancer), and C22 (liver cancer). We excluded data for which information on sex and/or age was missing (*N* = 2,309, 0.7%). We calculated the number of cancer-specific deaths per age category and for men and women separately. To obtain the number of cancer deaths that can be attributed to alcohol, we multiplied the disease-specific mortality numbers with the PAF of alcohol.

### Calculation of DALYs attributable to alcohol

Disability-adjusted life years (DALYs) is a composite measure that accounts both for morbidity (years lived with disability (YLDs)) and premature mortality (years of life lost (YLLs)), to express health loss in the population. YLLs are estimated by multiplying the estimated number of deaths by age with life expectancy at that age. We multiplied the number of deaths per age category, as derived from the vital registration data, with the life expectancy for Argentina per age category according to estimations from the World Health Organization (WHO) [27]. We derived the YLDs from the Global Health Data exchange, using the Global Burden of Disease (GBD) results tool [28], available from http://ghdx.healthdata.org/gbd-results-tool*.* Following GBD methodology, YLDs were calculated by multiplying disease prevalence by a disability weight [29]. A disability weight quantifies the severity of health loss associated with that disease, ranging on a scale from 0 (perfect health) to 1 (death) [30]. Since cancer has different phases, for which different disability weights are appropriate (e.g., the controlled phase of cancer has a lower disability weight than the terminal phase of cancer), specific disability weights were multiplied with the prevalence of each of the following four phases, or sequelae: 1) diagnosis and primary therapy phase, 2) controlled phase, 3) metastatic phase, and 4) terminal phase. Finally, YLLs and YLDs were summed to obtain DALYs, stratified by sex and age category. We calculated the number of DALYs that can be attributed to alcohol consumption by multiplying it with the PAF of alcohol.

We quantified disease burden stratified by alcohol consumption category. Heavier drinkers often have a higher risk for developing cancer as compared to light-to-moderate drinkers, and some cancer types are associated with heavy drinking only. However, on a population level there are more moderate drinkers than heavy drinkers. Therefore, we calculated for which exposure level the alcohol-attributable cancer burden was highest. Additionally, we explored three hypothetical scenarios to analyze which changes in alcohol consumption levels would account for the greatest reduction in alcohol-attributable cancer burden: 1) heavy drinkers become moderate drinkers, 2) moderate drinkers become light drinkers, and 3) moderate drinkers become light drinkers and heavy drinkers become moderate drinkers.

For three additional types of cancer (melanoma, pancreas, and prostate cancer) the meta-analysis reported a possible association with alcohol consumption. More recent evidence supports this claim [31–33]. Therefore, as a sensitivity analysis, we additionally included numbers of alcohol-attributable deaths and DALYs for melanoma (ICD-10 code: C43), pancreas (C25), and prostate cancer (C61) to estimate what the additional alcohol-attributable burden of cancer would be if these associations were to be legitimate.

## Results

In 2018, 53% of the Argentinean population consumed alcohol, this proportion was higher in men (66%) than in women (42%). Heavy drinkers (> 50 g/day) accounted for 2.4% of the population (in men 4.5% and in women 0.5%), moderate drinkers (12.6 – 50 g/day) accounted for 12.9% of the population (men: 19% and women: 7.2%). Proportion of heavy drinkers decreased with age, while abstention from alcohol was more prevalent in the higher age groups (Supplementary Table 2).

**Fig. 1:**
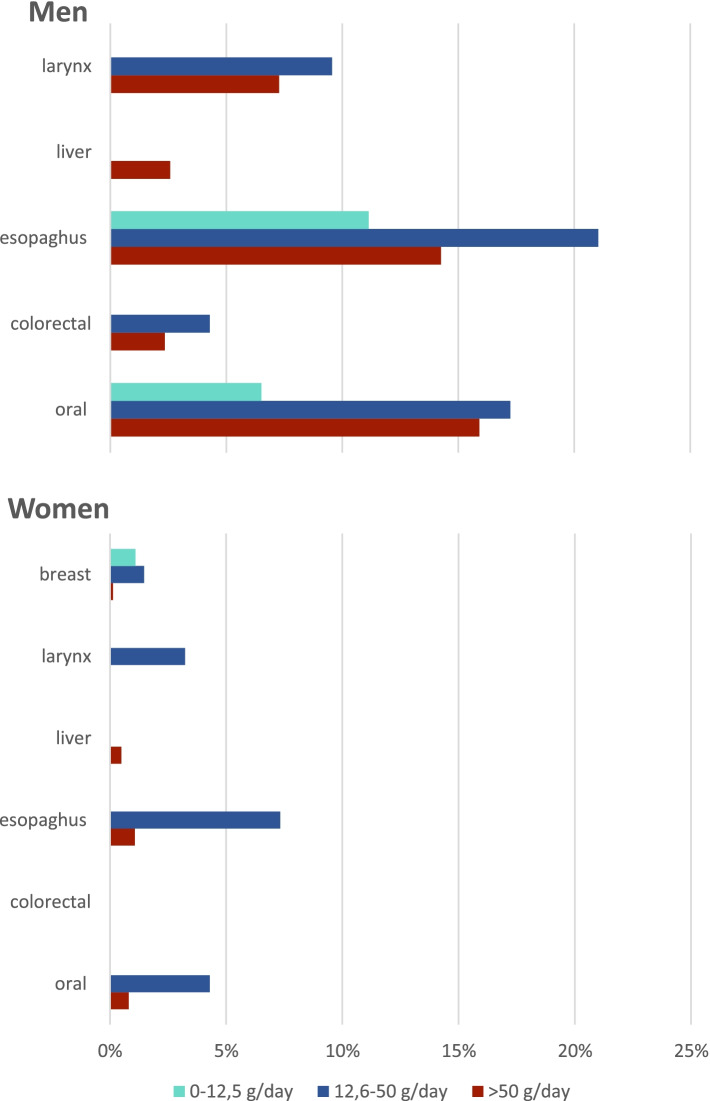
Proportion of cancer-specific deaths attributable to alcohol consumption in men and women, stratified by consumption level

The total number of deaths in 2018 in Argentina was 334,514, of which 60,764 (18%) were due to cancer. The six cancer types of interest in this study accounted for 18,255 (5%) of the total mortality cases (Supplementary Fig. 1). Mortality numbers were highest in the highest age categories (Supplementary Fig. 2). Alcohol consumption caused 1,387 deaths, this is 7.6% of the total number of deaths for these six types of cancer and 2.3% of the number of deaths for all cancers. Alcohol-attributable cancer mortality was higher in men (*N* = 1,166, 16% of deaths from the six types of cancer, 3.7% of deaths from all cancers) than in women (*N* = 221, 2% of six cancers, 0.8% of all cancers), in all age categories (Table 1 and Supplementary Fig. 3). In absolute numbers, most deaths attributable to alcohol consumption were found for esophageal cancer in men and breast cancer in women (Supplementary Fig. 4). However, the proportion of alcohol-attributable deaths was highest for esophageal cancer (45% in men and 8% in women) (Table 1). Most deaths (*N* = 713, 51%) attributable to alcohol consumption were found in the moderate consumption category (12.6 – 50 g/day) (Fig. 1).

**Table 1:** Number of cancer-specific deaths attributable to alcohol consumption

	MEN								WOMEN							
	**All alcohol consumption**		**0.1 – 12.5 g/day (light)**		**12.6 – 50 g/day (moderate)**		** > 50 g/day (heavy)**		**All alcohol consumption**		**0.1 – 12.5 g/day (light)**		**12.6 – 50 g/day (moderate)**		** > 50 g/day (heavy)**	
	*N*	PAF	*N*	PAF	*N*	PAF	*N*	PAF	*N*	PAF	*N*	PAF	*N*	PAF	*N*	PAF
***All selected cancer types***	1166	16%	167	2%	573	8%	426	6%	221	2%	63	0.6%	139	1%	19	0.2%
*Oral cavity and pharynx*	236	40%	39	7%	103	17%	95	16%	14	5%	0	0%	12	4%	3	1%
*Breast cancer*	-	-	-	-	-	-	-	-	155	3%	63	1%	84	1%	8	0.1%
*Esophageal cancer*	533	46%	128	11%	242	21%	164	14%	46	8%	0	0%	40	7%	8	1%
*Colorectal cancer*	260	7%	0	0%	168	4%	92	2%	0	0%	0	0%	0	0%	0	0%
*Liver cancer*	29	3%	0	0%	0	0%	29	3%	4	0.5%	0	0%	0	0%	4	0.5%
*Larynx cancer*	109	17%	0	0%	62	10%	47	7%	4	3%	0	0%	4	3%	0	0%

Number of alcohol-attributable deaths for each type of cancer, stratified by sex and alcohol consumption category. The denominator for the population attributable fraction is the total number of deaths due to that specific type of cancer. E.g., 7% of all deaths due to colorectal cancer in men can be attributed to alcohol consumption

*PAF*, population attributable fraction

In 2018, the total number of DALYs due to cancer was 1,137,397**.** Alcohol consumption caused 23,948 DALYs, which is 7% of all DALYs due to the six selected cancer types and 2.1% of DALYs due to all cancers. Again, alcohol consumption caused more DALYs in men (*N* = 18,904, 16% of six cancers, 3.7% of all cancers) than in women (*N* = 5,045, 2% of six cancers, 0.8% of all cancers). Similar to the mortality numbers, most alcohol-attributable DALYs (*N* = 12,059, 50%) were found in the moderate consumption category (Table 2).

**Table 2:** Number of cancer-specific DALYs attributable to alcohol consumption

	MEN								WOMEN							
	**All alcohol consumption**		**0.1 – 12.5 g/day (light)**		**12.6 – 50 g/day (moderate)**		** > 50 g/day (heavy)**		**All alcohol consumption**		**0.1 – 12.5 g/day (light)**		**12.6 – 50 g/day (moderate)**		** > 50 g/day (heavy)**	
	*N*	PAF	*N*	PAF	*N*	PAF	*N*	PAF	*N*	PAF	*N*	PAF	*N*	PAF	*N*	PAF
***All selected cancer types***	18,903	16%	2911	2%	9177	8%	6815	6%	5038	2%	1621	0.7%	2878	1%	538	0.2%
*Oral cavity and pharynx*	4478	39%	800	7%	1912	17%	1767	15%	275	5%	0	0%	218	4%	57	1%
*Breast cancer*	-	-	-	-	-	-	-	-	3801	3%	1621	1%	1930	1%	250	0.2%
*Esophageal cancer*	8126	46%	2111	12%	3600	20%	2415	14%	799	8%	0	0%	665	7%	134	1%
*Colorectal cancer*	4095	6%	0	0%	2658	4%	1437	2%	0	0%	0	0%	0	0%	0	0%
*Liver cancer*	436	3%	0	0%	0	0%	436	3%	97	0.7%	0	0%	0	0%	97	0.7%
*Larynx cancer*	1768	16%	0	0%	1007	9%	761	7%	66	3%	0	0%	66	3%	0	0%

Number of alcohol-attributable DALYs for each type of cancer, stratified by sex and alcohol consumption category. The denominator for the population attributable fraction is the total number of DALYs due to that specific type of cancer. E.g., 6% of all DALYs due to colorectal cancer in men can be attributed to alcohol consumption

*PAF*, population attributable fraction

Inclusion of melanoma, pancreas, and prostate cancer resulted in 186 additional attributable deaths and 2,677 additional attributable DALYs.

### Hypothetical scenarios

If heavy drinkers would shift to the moderate consumption category, 338 deaths and 5,498 DALYs would be prevented, a reduction of 24% in deaths and 23% in DALYs. However, a greater reduction of alcohol-attributable cancer burden would be achieved if moderate drinkers would shift to the light drinking category: almost half of all alcohol-attributable deaths and DALYs would be prevented (639 deaths (46%) and 10,853 DALYs (45%)). In this last scenario, if heavy drinkers would additionally shift to the moderate consumption category, this would prevent an additional 105 deaths and 4,523 DALYs. Therefore, in this final scenario, 54% of alcohol-attributable deaths and 64% of alcohol-attributable DALYs would be prevented.

## Discussion

In 2018, half of the population of Argentina reported to consume alcohol on a regular basis. A total number of 1,387 deaths and 23,948 DALYs related to cancer were attributable to alcohol consumption, accounting for 2.3% of the total cancer-related deaths and 2.1% of total cancer-related DALYs. Alcohol-attributable cancer mortality and disability was higher in men (3.7%) than in women (0.8%). Half of all deaths and DALYs attributable to alcohol consumption were found in the moderate consumption category (12,6 – 50 g/day). The greatest health gain would be achieved if all moderate drinkers would reduce their alcohol consumption to light drinking.

### Strengths and limitations

One of the strengths of this study is that we collected data from several Argentinean sources, to ensure representativeness of results for the Argentinean population. Furthermore, we used the most comprehensive meta-analysis so far on the association between alcohol consumption and various types of cancer. This research provided us with risk estimates stratified by alcohol consumption category, which allowed us to make comparisons between consumption levels.

At the same time, the main limitations also stem from the underlying data sources: the meta-analysis considered alcohol consumption between 12.6 and 50 g/day moderate drinking for both men and women, while the most recent alcohol consumption guidelines consider everyone who drinks > 2 units (= 28 g, in men) or > 1 unit (= 14 g, in women) daily a heavy drinker [9]. Therefore, this moderate drinking category in fact partly consists of heavy drinkers, which causes an overestimation of alcohol-attributable cancer burden for true moderate drinkers. However, since there exists a linear, dose–response, relationship between amount of alcohol consumed and risk of cancer, it is likely that shifting from moderate to light drinking still would cause a great reduction in disease burden [34]. Moreover, the limited number of women in the heavy drinking category (> 50 g/day) might have affected the precision of the risk estimates for heavy drinking, leading to non-significant associations in for example colorectal and larynx cancer. This might have led to an underestimation of the total alcohol-attributable cancer burden in women. Finally, the meta-analysis did not provide risk estimates for binge drinking, which also might have led to an underestimation of alcohol-attributable cancer burden.

The national risk factor survey provided us with information on prevalence of alcohol consumption levels in Argentina, but relied exclusively on self-reported, prone to willingness-to-please data and did not take into account unrecorded alcohol consumption. Moreover, we used data from the most recent survey and therefore did not consider the time interval between consumption of alcohol and development of cancer. Alcohol consumption levels have decreased in Argentina over the years—from over 15 L of annual pure alcohol consumption per capita in 1980 to around 9 L at present [2]—and we did not account for the higher levels of alcohol consumption in the past. Those limitations could have led to an underestimation of the PAF of alcohol consumption and therefore to an underestimation of alcohol-attributable cancer burden.

Taken together, it is likely that the limitations of this study merely caused an underestimation of true alcohol-attributable cancer burden. Therefore, the calculations in this work should be regarded as conservative and figures might in fact be higher.

### Implications of findings

Especially in males, we noticed substantial differences in alcohol-attributable cancer deaths and DALYs for the various types of cancer: especially for cancers of the upper digestive track (oral cavity, esophagus) the proportion of deaths and DALYs attributable to alcohol was quite high: 40–46%, compared to for example 3% of the deaths and DALYs in liver cancer. Overall, alcohol caused 2.3% of all cancer deaths in the year 2018. This is slightly lower than in the neighboring countries Chile [21] and Brazil [22] (respectively 3.1 and 4.5%), while similar methodology was used. We cannot entirely explain this difference, although differences in distribution of consumption levels in the population could be one of the reasons (e.g., in the Brazilian study, 25% of the male participants was heavy drinker). Praud et al. [20] estimated the percentage alcohol-attributable cancer deaths at 4% in the world region of the Americas, compared to 5.8% globally. This study also took unrecorded alcohol consumption into account.

In previous research, alcohol-attributable burden of ischemic heart disease (IHD) and stroke in Argentina has been calculated. The beneficial effects of moderate alcohol consumption on IHD slightly outweighed the harmful effects of any alcohol consumption on stroke and of binge drinking on both disease categories: in 2011 there was a net benefit of overall saving of 488 deaths and 33,551 DALYs [35]. The present study reports detrimental effects only of alcohol consumption on risk of cancer, and therefore the burden of disease caused by alcohol becomes greater than the burden of disease prevented by alcohol. This is still an underestimation, since GBD data showed that globally, when considering all other diseases, road accidents, and other (un)intentional injury, alcohol-attributable burden of disease is much higher: according to GBD data 6% of all deaths in Argentina are attributable to alcohol consumption [1]. We can therefore conclude that the potential benefits of moderate alcohol consumption on disease burden are very limited compared to the overall harmful contribution of alcohol to total burden of disease.

Despite a lower relative risk of cancer in the moderate as compared to the heavy alcohol consumption category, most deaths and DALYs were attributable to moderate drinking since there are far more moderate than heavy drinkers in the population. When heavy drinkers would shift to moderate drinking, 24% of alcohol-attributable cancer deaths and DALY’s would be prevented. However, if moderate drinkers would shift to light drinking, this percentage would be 46%: almost half of all alcohol-attributable deaths and DALYs. This is the prevention paradox, which states that the greatest disease burden in the population is caused by the group at low-to-moderate risk and not by the group at high risk, because the low-to-moderate risk group is much larger in absolute number [23]. We do not assume the modelled scenarios to be realistic in the sense that all heavy drinkers would shift to moderate drinking, or all moderate drinkers would shift to light drinking following alcohol policy measures. We however do think that, in terms of public health policy, a population-wide strategy aimed to shift the population distribution of alcohol consumption levels would be more effective in reducing alcohol-related harm than targeting high-risk drinkers only [34]. Recently, the WHO has launched the global SAFER initiative [5], that provides five population-wide strategies (“best buys”) to reduce the harmful use of alcohol in the population, including: reducing availability of alcohol, increased taxation on alcohol-containing products, restrictions on alcohol advertising and promotion, better access to screening and brief interventions, and stricter legislation to prevent drink-driving. Implementation of such strategies on a public health level is known to be challenging, since it always involves several other players (political, economic) with conflicting interests [36]. Although in Argentina specific regulation exists (National Law No. 24,788), the norm is not sufficient for an effective control of the consumption of alcoholic beverages in the adolescent population since it does not comply with the international standards. Argentina already has implemented some of the SAFER measures in the past recent years but could certainly benefit from additional strategies to further reduce overall consumption of alcohol.

## Conclusion

Alcohol consumption accounted for 2.3% of all cancer deaths and 2.1% of all cancer DALYs in Argentina in 2018. Most deaths and DALYs were attributable to moderate alcohol consumption, since heavy drinkers only make up for a small proportion of the population. This calls for implementation of population-wide strategies to reduce harmful use of alcohol in the general population and its impact on the total burden of disease.

## Supplementary Information

**Additional file 1: Supplementary table 1.** Cancer site-specific risk estimates per alcohol consumption category. **Supplementary table 2. **Prevalence of alcohol consumption patterns in the general population of Argentina in 2018, stratified by age and sex. **Supplementary figure 1. **Venn diagram of total number of deaths in Argentina in 2018, including those due to cancer, those due to types of cancer associated with alcohol consumption and the deaths that can be attributed to alcohol consumption. **Supplementary figure 2. **Number of deaths due to the six selected cancer types, per age category. Vital registration data from 2018, Directorate of Health Statistics and Information (DEIS) of the Argentine government [25]. **Supplementary figure 4.** Distribution of alcohol-attributable deaths per type of cancer for men (left) and women (right) in absolute numbers.

## Declarations

### Availability of data and materials

All data are publicly available

1. Relative risks (RRs): obtained from study by Bagnardi et al., https://doi.org/10.1038/bjc.2014.579
2. Data 4^th^ National Risk Factor Survey: https://www.indec.gob.ar/indec/web/Institucional-Indec-BasesDeDatos-2\
3. Vital registration data, obtained from the Directorate of Statistics and Health Information (DEIS): https://www.argentina.gob.ar/salud/deis/datos
4. Life expectancy Argentina from Global Health Repository of the World Health Organization: https://apps.who.int/gho/data/node.main.LIFECOUNTRY?lang=en
5. Data for calculation DALYs from Global Health Data Exchange: http://ghdx.healthdata.org/gbd-results-tool
