# Supplementary material for: Alcohol-attributable burden of cancer in Argentina
Source: BMC Public Health. 2022 Jan 18;22:124. doi: 10.1186/s12889-022-12549-7 (PMC8764501; doi:10.1186/s12889-022-12549-7)
Supplement: Supplementary file 1 — Additional file 1: Supplementary table 1. Cancer site-specific risk estimates per alcohol consumption category. Supplementary table 2. Prevalence of alcohol consumption patterns in the general population of Argentina in 2018, stratified by age and sex. Supplementary figure 1. Venn diagram of total number of deaths in Argentina in 2018, including those due to cancer, those due to types of cancer associated with alcohol consumption and the deaths that can be attributed to alcohol consumption. Supplementary figure 2. Number of deaths due to the six selected cancer types, per age category. Vital registration data from 2018, Directorate of Health Statistics and Information (DEIS) of the Argentine government [25]. Supplementary figure 4. Distribution of alcohol-attributable deaths per type of cancer for men (left) and women (right) in absolute numbers. [file 12889_2022_12549_MOESM1_ESM.docx]

**Supplementary materials**

**Alcohol-attributable burden of cancer in Argentina.**

I.A.T. van de Luitgaarden et al.

| **List of Supplementary Materials** | **Page** |
| --- | --- |
| Supplementary Table 1. Cancer site-specific risk estimates per alcohol consumption category. | 2 |
| Supplementary Table 2. Prevalence of alcohol consumption patterns in the general population of Argentina in 2018, stratified by age and sex. | 4 |
| Supplementary Figure 1. Venn diagram of total number of deaths in Argentina in 2018 | 5 |
| Supplementary Figure 2. Number of deaths due to the six selected cancer types, per age category. | 6 |
| Supplementary Figure 3. Number of alcohol-attributable deaths, stratified by age category and sex. | 6 |
| Supplementary Figure 4. Distribution of alcohol-attributable deaths per type of cancer for men and women in absolute numbers | 7 |

**Supplementary table 1. Cancer site-specific risk estimates per alcohol consumption category.**

| **Cancer type** | **Alcohol consumption category** | **RR (men)** | **RR (women)** | **Analyses adjusted for** |
| --- | --- | --- | --- | --- |
|  |  |  |  |  |
| **Oral cavity and pharynx** | Non-drinker | Reference | Reference | *Age, Sex, Smoking* |
|  | 0.1 – 12,5 g/day | **1.20 (1.06 – 1.35)** | 1.00 (0.78 – 1.27) |  |
|  | 12,6 – 50 g/day | **2.01 (1.69 – 2.40)** | **1.67 (1.25 – 2.22)** |  |
|  | >50 g/day | **5.33 (4.28 – 6.63)** | **5.70 (3.75 – 8.66)** |  |
| **Breast cancer (in women only)** | Non-drinker | - | Reference | *Age, Family history, Parity, Menopausal status, OC use/ HRT* |
|  | 0.1 – 12,5 g/day | - | **1.04 (1.01 – 1.07)** |  |
|  | 12,6 – 50 g/day | - | **1.23 (1.19 – 1.28)** |  |
|  | >50 g/day | - | **1.61 (1.33 – 1.94)** |  |
| **Esophageal cancer** | Non-drinker | Reference | Reference | *Age, Sex, Smoking* |
|  | 0.1 – 12,5 g/day | **1.39 (1.11 – 1.74)** | 1.14 (0.87 – 1.49) |  |
|  | 12,6 – 50 g/day | **2.25 (1.78 – 2.85)** | **2.18 (1.42 – 3.35)** |  |
|  | >50 g/day | **4.69 (3.49 – 6.31)** | **8.32 (2.95 – 23.45)** |  |
| **Colorectal cancer** | Non-drinker | Reference | Reference | *Age, sex, BMI or physical activity* |
|  | 0.1 – 12,5 g/day | 1.05 (0.95 – 1.16) | 0.95 (0.89 – 1.01) |  |
|  | 12,6 – 50 g/day | **1.21 (1.11 – 1.32)** | 1.07 (0.99 – 1.16) |  |
|  | >50 g/day | **1.53 (1.30 – 1.80)** | 1.24 (0.68 – 2.25) |  |
| **Liver cancer** | Non-drinker | Reference | Reference | *Age, Sex, Liver Disease, BMI or Diabetes* |
|  | 0.1 – 12,5 g/day | 1.05 (0.84 – 1.32) | 0.81 (0.59 – 1.12) |  |
|  | 12,6 – 50 g/day | 1.08 (0.88 – 1.32) | 1.24 (0.88 – 1.75) |  |
|  | >50 g/day | **1.59 (1.21 – 2.09)** | **3.89 (1.60 – 9.48)** |  |
| **Larynx cancer** | Non-drinker | Reference | Reference | *Age. Sex, Smoking* |
|  | 0.1 – 12,5 g/day | 0.85 (0.61 – 1.19) | 0.89 (0.62 – 1.29) |  |
|  | 12,6 – 50 g/day | 1.50 (1.23 – 1.83) | 1.59 (1.06 – 2.38) |  |
|  | >50 g/day | 2.77 (2.15 – 3.57) | 1.55 (0.45 – 5.34) |  |
| **Pancreas cancer*** | Non-drinker | Reference | Reference | *Age. Sex, Smoking* |
|  | 0.1 – 12,5 g/day | 0.98 (0.86 – 1.11) | 0.93 (0.86 – 1.01) |  |
|  | 12,6 – 50 g/day | **1.08 (1.00 – 1.15)** | 1.04 (0.93 – 1.17) |  |
|  | >50 g/day | **1.16 (1.06 – 1.27)** | 1.17 (0.98 – 1.40) |  |
| **Prostate cancer†** | Non-drinker | Reference | - | *Age, Family history, Race* |
|  | 0.1 – 12,5 g/day | **1.04 (1.01 – 1.08)** | - |  |
|  | 12,6 – 50 g/day | **1.06 (1.01 – 1.11)** | - |  |
|  | >50 g/day | **1.18 (1.10 – 1.27)** | - |  |
| **Melanoma‡** | Non-drinker | Reference | Reference | *Age, Family history, Sun exposure, Number of nevi* |
|  | 0.1 – 12,5 g/day | 1.19 (0.82 – 1.72) | **1.25 (1.13 – 1.38)** |  |
|  | 12,6 – 50 g/day | 1.32 (0.90 – 1.92) | **1.27 (1.14 – 1.43)** |  |
|  | >50 g/day | Not evaluable | Not evaluable |  |

Source: RRs obtained from Bagnardi et al (17). risk estimates in bold reached statistical significance (p < 0.05).

*included in sensitivity analyses, based on new evidence by Zhao et al. Risk estimates partly obtained from Zhao et al. (30)

† included in sensitivity analysis, based on new evidence by Wang et al. (31)

‡ included in sensitivity analysis, based on new evidence by Gandini et al. (32)

Abbreviations: BMI; body mass index, OC; oral contraceptive, HRT; hormonal replacement therapy, RR; relative risk.

**Supplementary table 2. Prevalence of alcohol consumption patterns in the general population of Argentina in 2018, stratified by age and sex.**

|  | Non-drinkers | 0 – 12.5 g/day (light) | 12.6 – 50 g/day (moderate) | > 50 g/day (heavy) |
| --- | --- | --- | --- | --- |
| MEN |  |  |  |  |
| **18-29 y** | 30.5% | 44.0% | 19.5% | 6.0% |
| **30-39 y** | 30.7% | 48.7% | 17.6% | 3.0% |
| **40-49 y** | 31.2% | 46.7% | 17.7% | 4.4% |
| **50-59 y** | 34.7% | 42.1% | 18.8% | 4.5% |
| **60-69 y** | 41.5% | 34.6% | 19.9% | 4.0% |
| **≥70 y** | 45.3% | 27.1% | 22.9% | 4.8% |
| WOMEN |  |  |  |  |
| **18-29 y** | 49.2% | 40.0% | 9.9% | 0.9% |
| **30-39 y** | 54.9% | 37.5% | 7.3% | 0.3% |
| **40-49 y** | 57.8% | 36.5% | 5.0% | 0.7% |
| **50-59 y** | 65.3% | 29.5% | 4.9% | 0.3% |
| **60-69 y** | 63.4% | 29.6% | 6.8% | 0.2% |
| **≥70 y** | 69.7% | 23.0% | 7.3% | 0.1% |

Data obtained from the 4th national risk factor survey, 2018 (24).


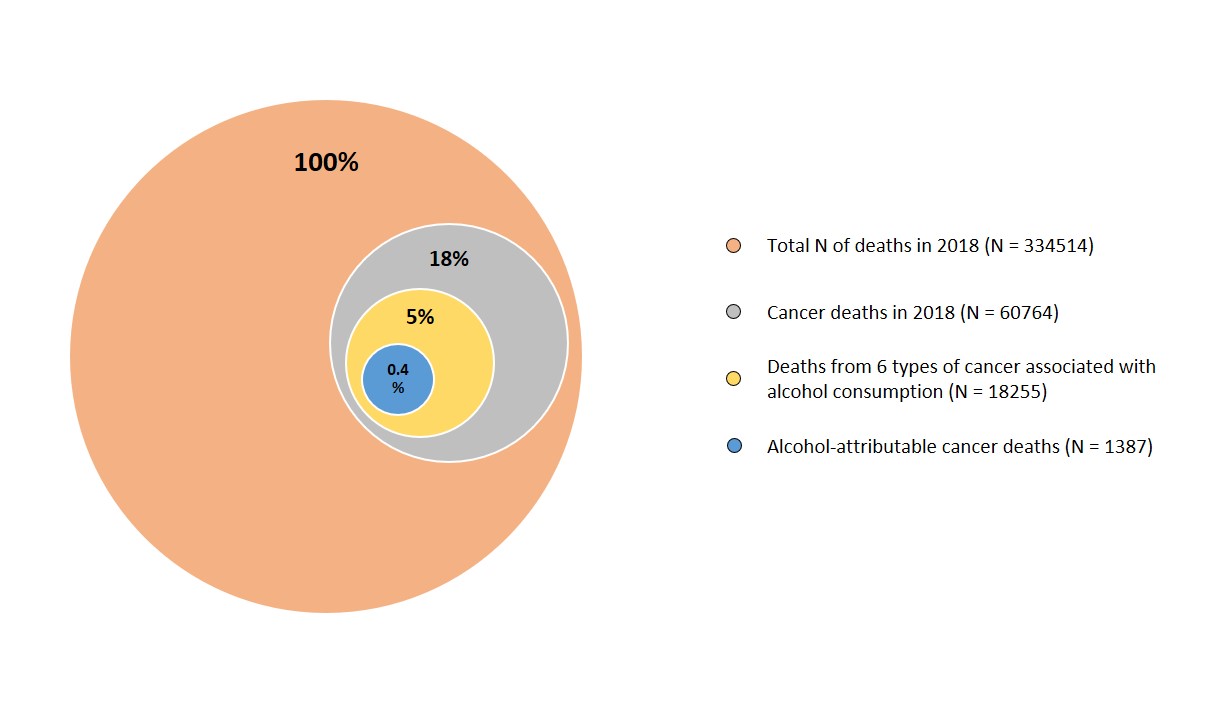


**Supplementary figure 1.** Venn diagram of total number of deaths in Argentina in 2018, including those due to cancer, those due to types of cancer associated with alcohol consumption and the deaths that can be attributed to alcohol consumption.

**Supplementary figure 2.** Number of deaths due to the six selected cancer types, per age category. Vital registration data from 2018, Directorate of Health Statistics and Information (DEIS) of the Argentine government (25).

**Supplementary figure 3.** Number of alcohol-attributable deaths, stratified by age category and sex.

|  |  |
| --- | --- |

**Supplementary figure 4**. Distribution of alcohol-attributable deaths per type of cancer for men (left) and women (right) in absolute numbers
